# miR-6402 targets *Bmpr2* and negatively regulates mouse adipogenesis

**DOI:** 10.1080/21623945.2025.2474114

**Published:** 2025-03-03

**Authors:** Malaz Elsheikh, Tomomi Sano, Akiko Mizokami, Yusuke Nakatsu, Tomoichiro Asano, Takashi Kanematsu

**Affiliations:** a Department of Cell Biology, Aging Science, and Pharmacology, Division of Oral Biological Sciences, Faculty of Dental Science, Kyushu University, Fukuoka, Japan; b OBT Research Center, Faculty of Dental Science, Kyushu University, Fukuoka, Japan; c Department of Biological Chemistry, Institute of Biomedical and Health Sciences, Hiroshima University, Hiroshima, Japan

**Keywords:** Adipocyte, adipogenesis, bone morphogenetic protein receptor type 2, microRNA, miR-6402

## Abstract

Obesity is characterized by macrophage infiltration into adipose tissue. White adipose tissue remodelling under inflammatory conditions involves both hypertrophy and adipogenesis and is regulated by transcription factors, which are influenced by bone morphogenetic protein (BMP) signalling. MicroRNAs (miRNAs) regulate gene expression and are involved in obesity-related processes such as adipogenesis. Therefore, we identified differentially expressed miRNAs in the epididymal white adipose tissue (eWAT) of mice fed a normal diet (ND) and those fed a high-fat diet (HFD). The expression of miR-6402 was significantly suppressed in the inflamed eWAT of HFD-fed mice than in ND-fed mice. Furthermore, *Bmpr2*, the receptor for BMP4, was identified as a target gene of miR-6402. Consistently, miR-6402 was downregulated in the inflamed eWAT of HFD-fed mice and in 3T3-L1 cells (preadipocytes) and differentiated 3T3-L1 cells (mature adipocytes) , and BMPR2 expression in these cells was upregulated. Adipogenesis was induced in WAT by BMP4 injection (*in vivo*) and in 3T3-L1 cells by BMP4 stimulation (*in vitro*), both of which were inhibited by miR-6402 transfection. Inflamed eWAT showed higher expression of BMPR2 and the adipogenesis markers C/EBPβ and PPARγ, which was suppressed by miR-6402 transfection. Our findings suggest that miR-6402 is a novel anti-adipogenic miRNA that combats obesity by inhibiting the BMP4/BMPR2 signalling pathway and subsequently reducing adipose tissue expansion.

## Introduction

According to the recent World Obesity Atlas 2023 report from the World Obesity Federation, over half of the global population will become overweight or obese by 2035 if preventive actions against obesity are not taken. Obesity can lead to various health complications such as type II diabetes mellitus, dyslipidemia, hypertension, cardiovascular diseases, and several major cancers [1,2]. Therefore, development of new and effective therapeutic interventions for obesity remains an urgent and unmet medical need.

The progression of obesity is characterized by marked infiltration of macrophages, the primary source of the proinflammatory cytokine tumour necrosis factor (TNF)-α, into the adipose tissue and dynamic expansion of the adipose tissue through adipocyte hypertrophy and/or hyperplasia, resulting in the development of obesity-related complications [3]. Dysregulation of adipogenesis, which is defined by preadipocyte differentiation into mature adipocytes or fat cells, also contributes to the expansion of adipose tissue mass [4]. Therefore, a better understanding of the cellular biology of adipogenesis may yield novel strategies for distinguishing obesity from other metabolic diseases.

Adipogenesis consists of two stages: commitment of mesenchymal stem cells to form preadipocytes and differentiation of preadipocytes into mature adipocytes [5]. Different signalling pathways, including bone morphogenetic protein (BMP) signalling, regulate adipogenic processes. BMP4 is important for the induction of adipogenesis and functions by binding to BMP receptor type I (BMPR1) and type II (BMPR2) [6]. BMP4-treated C3H10T1/2, a mouse pluripotent stem cell line, has been shown to differentiate into adipocytes with increased expression of CCAAT enhancer binding protein (C/EBP) family proteins and peroxisome proliferator-activated receptor gamma (PPARγ), which are critical transcription factors in adipogenesis [7,8]

MicroRNAs (miRNAs) are short single-stranded, noncoding RNA molecule that binds to the 3′ untranslated region (3′-UTR) of messenger RNA, regulating gene expression by either suppressing translation or inducing degradation. miRNAs appear to play regulatory roles in numerous biological processes linked to obesity, including adipogenesis [9]. Emerging evidence has revealed the involvement of miRNAs in the pathogenesis of numerous diseases. The relationship between obesity and miRNAs has been investigated by exploring miRNA expression patterns in the serum and adipose tissue in animal models [10] and patients [11]. Therefore, miRNAs are potential therapeutic targets for the treatment of obesity.

In this study, we used our previously analysed microarray data to identify a possible miRNA that regulates obesity by comparing the differential expression of miRNAs in epididymal white adipose tissue (eWAT) between mice fed a normal diet (ND) and those fed a high-fat diet (HFD). miR-6402 was downregulated in inflamed adipose tissues, and the expression of *Bmpr2* was downregulated by exogenous expression of miR-6402. Importantly, reduced expression of miR-6402 in inflamed adipocytes promoted BMP4/BMPR2-mediated adipogenesis. To our knowledge, this is the first report showing that the downregulation of miR-6402 during obesity promotes adipogenesis.

## Results

### miR-6402 is suppressed in the obese mouse adipose tissues and TNF-α-stimulated adipocytes

**Figure 1.**
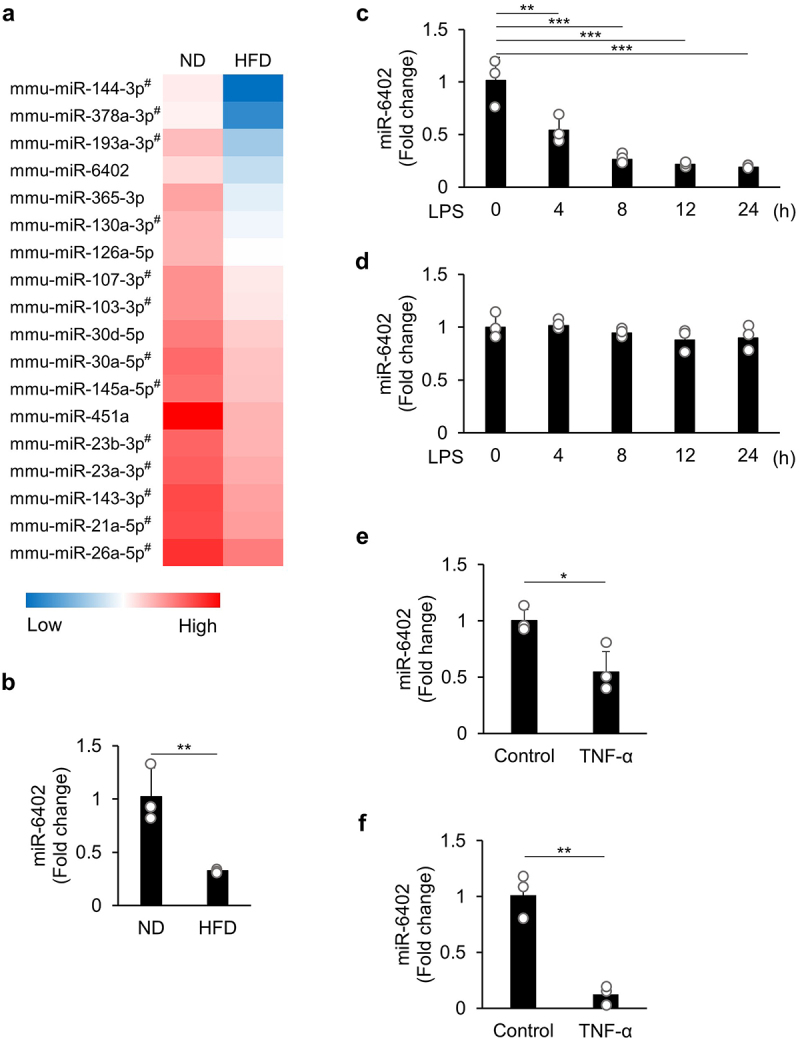
miR-6402 is suppressed in the obese mouse adipose tissues and tnf-α-stimulated adipocytes. (a) Heatmap of downregulated miRNAs in eWAT after HFD feeding in comparison with the findings in nd-fed mice. (b–f) qPCR analyses were performed to assess miR-6402 expression in eWAT (b), differentiated 3T3-L1 cells co-cultured with RAW264.7 cells (c), or RAW264.7 cells (d) stimulated with LPS for the indicated times, and undifferentiated (e) or differentiated (f) 3T3-L1 cells stimulated with tnf-α (5 ng/mL) for 4 h. The bar graphs represent the mean ± S.D. (*n* = 3 for each group). *p < 0.05, **p < 0.01, ***p < 0.001 between the indicated bars of the two groups (b, e, and f; Student’s *t*-test; c and d, Tukey – Kramer HSD test).

To identify a novel miRNA that regulates obesity-induced inflammation in the adipose tissue, we used data from a previously performed microarray analysis with eWATs from ND- and HFD-fed mice. Data on the body weight changes of the mice and the expression of inflammatory cytokines in the eWATs used are shown in the Supplementary Fig. S1a and b. The miRNA data are published in the NCBI Gene Expression Omnibus database (GSE216923) [12], and include the data for 100 highly expressed miRNAs in ND-fed mice with corresponding data from HFD-fed mice (Supplementary Fig. S2). Among these miRNAs, 18 miRNAs were identified as significantly suppressed in HFD-fed mice than in ND-fed mice (marked with asterisks in Supplementary Fig. S2) and arranged in descending order of reduction ratio (Figure 1(a)). Thirteen miRNAs were reported to have functions in obesity and are listed in Supplementary Table S1 (marked with a pound to the right of the miRNA name in Figure 1(a)). Since the first three listed miRNAs have been reported previously, we focused on miR-6402, which ranked third in terms of reduction ratio, and investigated its functions in the aetiology of obesity.

Using quantitative polymerase chain reaction (qPCR), we confirmed that miR-6402 expression levels were significantly lower in the eWAT of HFD-fed mice than in ND-fed mice (Figure 1(b)). We then examined the alterations in miR-6402 expression using differentiated 3T3-L1 cells co-cultured with RAW264.7 cells, an *in vitro* model of adipose tissue inflamed by lipopolysaccharide (LPS) stimulation. The expression of miR-6402 was significantly suppressed 4 h after the start of co-culture, and this suppression was maintained until the end of the experiment (Figure 1(c)). The expression of miR-6402 in LPS-stimulated RAW264.7 cells was also analysed but was not altered up to 24 h (Figure 1(d)). Furthermore, miR-6402 expression was very low in RAW264.7 macrophages compared to preadipocytes (3T3-L1) and differentiated 3T3-L1 adipocytes (Supplementary Fig. S1c). Therefore, miR-642 May function in response to obesity-induced eWAT inflammation in adipocytes. Since macrophages infiltrate adipocytes and release the inflammatory cytokine TNF-α during adipose inflammation, we stimulated 3T3-L1 preadipocytes and mature adipocytes with TNF-α and analysed the changes in miR-6402 expression. TNF-α treatment significantly reduced the expression of miR-6402 in both preadipocytes (Figure 1(e)) and mature adipocytes (Figure 1(f)).

### *Bmpr2* is the target of miR-6402 and is upregulated in inflamed adipocytes

**Figure 2.**
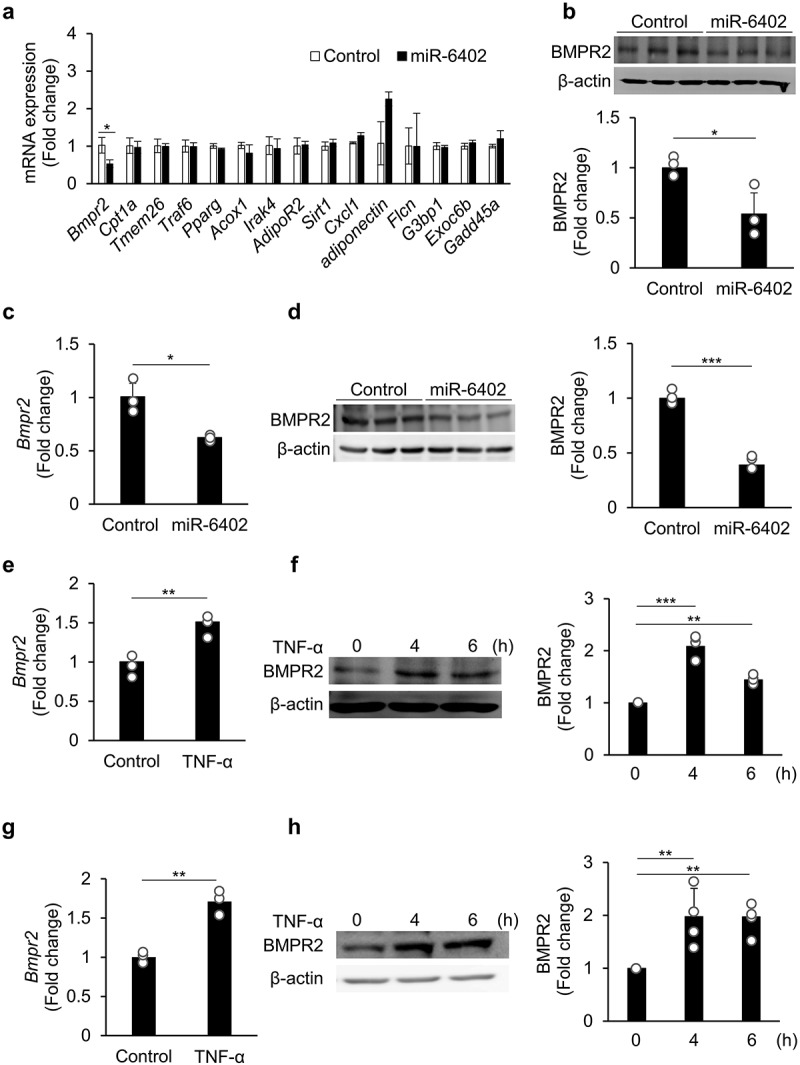
*Bmpr2* is the target of miR-6402 and is upregulated in inflamed adipocytes. Undifferentiated (a and b) and differentiated (c and d) 3T3-L1 cells were transfected with negative control microRNA (control) or miR-6402 mimics for 24 h. Significant expression of miR-6402 was observed in undifferentiated and differentiated 3T3-L1 cells (supplementary fig. S3a and b), indicating successful transfection of miR-6402. Expression of the indicated potential miR-6402 target genes predicted by TargetScan was measured by qPCR (a). BMPR2 expression in undifferentiated 3T3-L1 cells transfected with control and miR-6402 mimics was analysed by western blotting (b), BMPR2 expression in differentiated 3T3-L1 cells transfected with control and miR-6402 mimics was analysed by qPCR (c) and western blotting (d). BMPR2 expression was measured by qPCR (e and g) and western blotting (f and h) using undifferentiated (e and f) or differentiated (g and h) 3T3-L1 cells stimulated with tnf-α (5 ng/mL). β-actin was used as the loading control. Original whole-membrane and repeated images are shown in supplementary fig. S4, S5, S6 and S7. The bar graphs represent the mean ± S.D. (*n* = 3–4 for each group). *p < 0.05, **p < 0.01, ***p < 0.001 between the indicated bars of the two groups (a – e and g, Student’s *t*-test; f and h, Tukey – Kramer HSD test).

Next, we attempted to identify the target of miR-6402. Using TargetScan (release 7.2), 15 potential target genes of miR-6402 expressed in adipocytes were identified and are listed in Figure 2(a). Using qPCR, we analysed whether the mRNAs of these 15 genes were downregulated in 3T3-L1 cells transfected with miR-6402. *Bmpr2* expression was significantly suppressed in miR-6402-transfected 3T3-L1 cells than in the corresponding control, but the other genes showed no significant differences between the two groups (Figure 2(a)). We confirmed that BMPR2 protein expression was suppressed in miR-6402-transfected 3T3-L1 cells in comparison with that in control miRNA-transfected 3T3-L1 cells (Figure 2(b)). The expression of BMPR2 gene and protein was also suppressed in miR-6402-transfected differentiated 3T3-L1 cells compared to corresponding control cells (Figure 2(c,d)). Since *Bmpr2* may be a target of miR-6402, we investigated the expression of BMPR2. 3T3-L1 preadipocytes and mature adipocytes were stimulated with TNF-α, and BMPR2 expression was analysed. The gene and protein expression of BMPR2 was significantly upregulated in 3T3-L1 preadipocytes (Figure 2(e,f)) and mature adipocytes (Figure 2(g,h)) at 4 h (gene expression) or 4 and 6 h (protein expression) after stimulation, indicating that BMPR2 is the target of miR-6402 and that its expression is increased under inflammatory conditions.

### miR-6402 inhibits BMP4-induced adipogenesis

**Figure 3.**
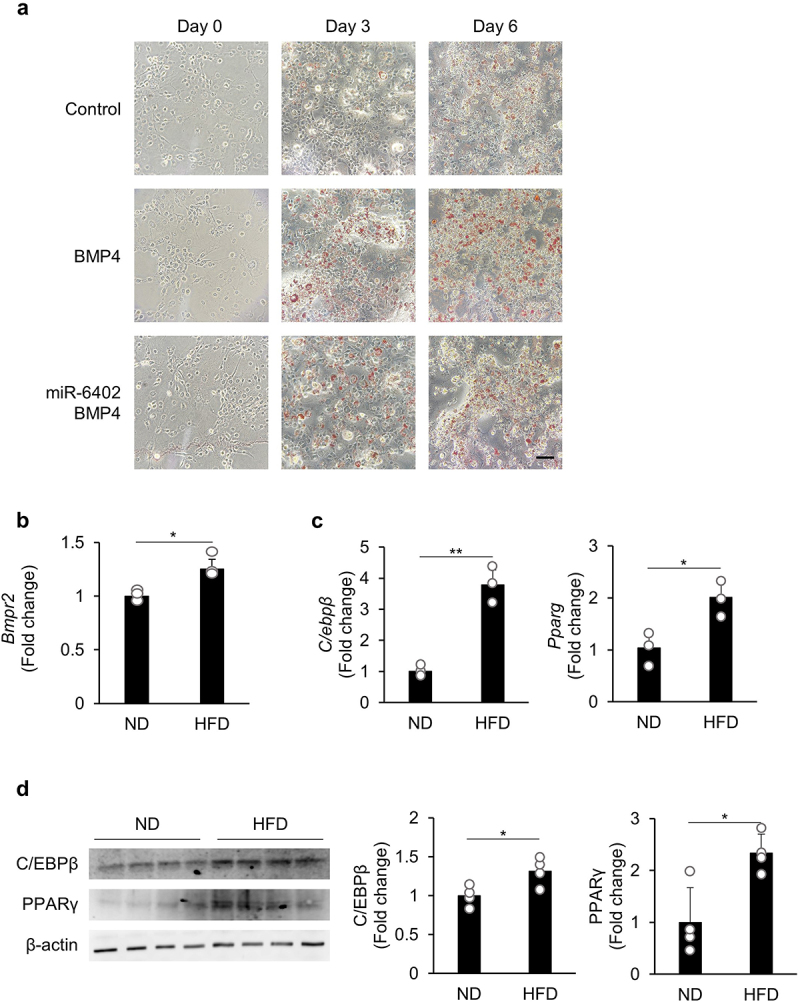
miR-6402 inhibits BMP4-induced adipogenesis. (a) Oil red O staining on day 0, 3, and 6. 3T3-L1 cells were cultured to 50–70% confluence and transfected with miR-6402 mimic. After 24 h, the cells were stimulated with a combination of differentiation media in the presence or absence of BMP4 (10 ng/mL). Scale bar: 100 µm. (b – d) expression of BMPR2, C/EBPβ, and PPARγ by qPCR (b and c) or western blotting (d) in eWAT from ND- or hfd-fed mice. Original whole-membrane images of (d) are shown in supplementary fig. S8. β-actin was used as the loading control. Data represent the mean ± SD (*n* = 3–4 for each group). *p < 0.05, **p < 0.01 (Student’s *t*-test).

BMP4 is a ligand of BMPR2 and plays a role in the early steps that promote the conversion of preadipocytes to undergo adipogenesis [13,14]. Therefore, we investigated the influence of miR-6402 on adipogenesis in 3T3-L1 cells by culturing the cells in a differentiation medium with or without BMP4. In the Oil Red O staining experiment, BMP4-promoted adipogenesis was suppressed by miR-6402 (Figure 3(a)). These data suggest that miR-6402 negatively regulates BMP4/BMPR2 signalling during adipogenesis.

Next, we investigated whether the expression of BMPR2, the receptor of BMP4, and adipogenesis were also enhanced in inflamed eWAT. Using the eWAT from HFD-fed mice, the expression of BMPR2 or the adipogenesis markers C/EBPβ (an early-stage marker) and PPARγ (a late-stage marker) were analysed by qPCR. *Bmpr2* levels were significantly higher in eWAT from HFD-fed mice than in eWAT from ND-fed mice (Figure 3(b)). Consistently, the expression of C/EBPβ and PPARγ was also significantly higher in the eWAT of HFD-fed mice than in that of ND-fed mice (Figure 3(c,d)), suggesting that the suppression of miR-6402 in inflamed eWAT (Figure 1(b)) upregulates BMPR2 expression, resulting in enhanced adipogenic signalling.

### miR-6402 inhibits BMP4-induced adipogenesis in vivo

**Figure 4.**
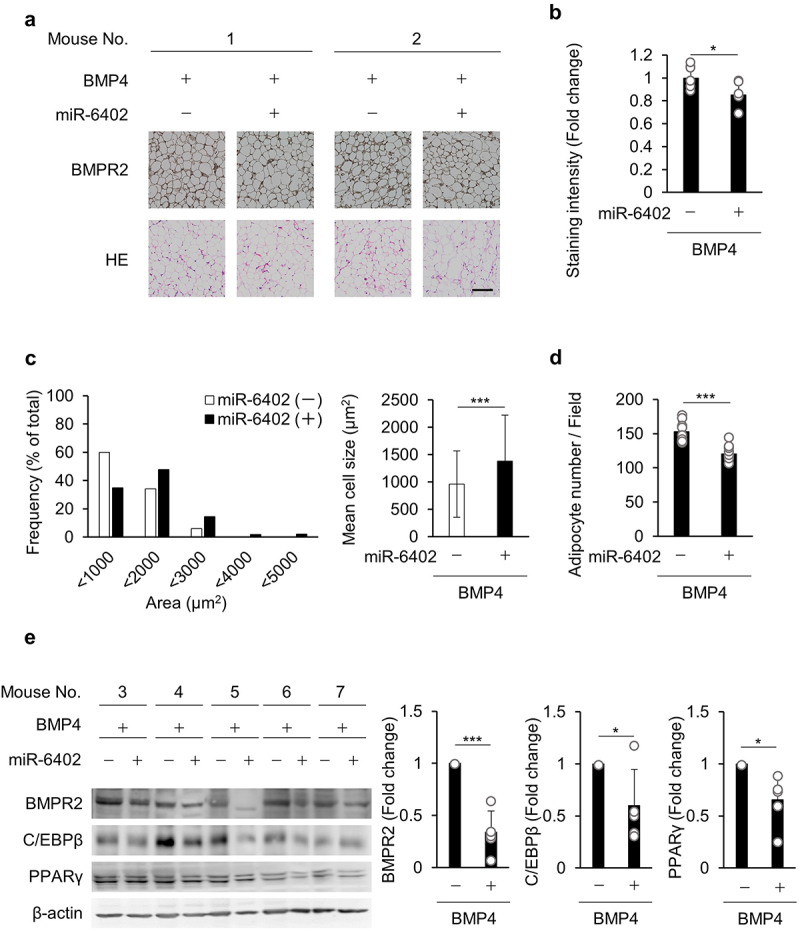
miR-6402 inhibits BMP4-induced adipogenesis *in vivo*. (a–e) BMP4 was injected into eWAT together with control or miR-6402 mimic. Two out of the seven mice were used for immunohistochemical staining of BMPR2 and hematoxylin-eosin staining (a; mouse No. 1 and 2), and the other five mice were used for analysis of BMPR2, C/EBPβ, and PPARγ expression by western blotting (e; mouse No. 3–7). Four different images from mice 1 and 2 (i.e. 8 images from two mouse eWAT samples) were obtained and analyzed for the immunohistochemical staining intensity of BMPR2 (b), frequency and mean cell size of adipocytes (c), and number of adipocytes (d). Representative image sets are shown in (a). Scale bar; 100 µm. Other images are shown in supplementary fig. S10. Western blotting results are shown on the far left in (e), and these analyzed data are shown as three bar graphs on the right. β-actin was used as a loading control. Original whole-membrane images are shown in supplementary fig. S11. The bar graphs represent the mean ± S.D. *p < 0.05, ***p < 0.001 between the indicated bar of two groups (Student’s *t*-test).

Finally, we examined the inhibitory effects of miR-6402 on adipogenesis *in vivo*. The combination of BMP4 and control or miR-6402 mimic was co-injected into the eWAT of the mice. At 24 h after injection, miR-6402 expression was obviously upregulated in the miR-6402-transfected eWAT compared to the corresponding control (Supplementary Fig. S9). Immunohistochemical staining of BMPR2 and haematoxylin-eosin (HE) staining were performed (Figure 4(a)). BMPR2 immunohistochemical staining intensity in the eWAT of the BMP4/miR-6402-injected side was significantly suppressed in comparison with that in the BMP4/control mimic-injected side (Figure 4(b)). In addition, BMP4 injection increased the number of small adipocytes, which was suppressed by miR-6402 co-injection, and the mean cell size was larger in eWAT injected with miR-6402 than in eWAT injected with the control mimic (Figure 4(c)). Consequently, the number of adipocytes in a single field was significantly reduced in eWAT injected with miR-6402 (Figure 4(d)). Consistently, BMPR2 levels were significantly downregulated in the miR-6402-injected eWAT than in the control, and the adipogenic markers C/EBPβ and PPARγ were downregulated in the miR-6402-injected eWAT (Figure 4(e)). Taken together, these findings suggest that miR-6402 suppresses BMP4-induced adipogenesis by inhibiting BMPR2 expression in mice.

## Discussion

Obesity and related metabolic complications are major health challenges due to excessive adipose hypertrophy, adipogenesis, or both [15]. In the present study, we identified miR-6402 as a novel obesity regulator that targets *Bmpr2* and inhibits BMP4/BMPR2-induced adipogenesis.

Several miRNAs whose expression is altered in obesity, in line with our heatmap results (Supplementary Fig. S2), have been reported to be associated with obesity and obesity-related complications. For example, the first miRNA listed in Figure 1(a), miR-144-3p, was found to impair the insulin signalling phenotype associated with obesity [16], and the second miRNA in the list, miR-378a-3p, was involved in the prevention of dietary betaine-mediated obesity [17]. Moreover, miR-193a-3p, the third listed miRNA, was found to influence insulin signalling in piglets [18]. Similarly, other miRNAs marked with pounds in Figure 1(a) have been reported to be associated with the regulation of obesity (Supplementary Table S1). In addition to these miRNAs, specific miRNAs that are involved in the regulation of adipogenesis have also been identified. Overexpression of miR-335 induces adipogenesis and is significantly upregulated in obese mice [19]. Adipogenesis can be impaired by miR-369-5p, whereas it is highly increased by miR-371 [20].

Similar to miR-6402, other anti-adipogenic miRNAs include miR-224-5p [21], miR-155 [22], and miR-145 [23]. PPARγ is known as an adipogenesis marker, and its function is essential for adipogenesis. miR-27a and miR-27b have been shown to function as anti-adipogenic regulators by targeting *Pparg* and preventing the induction of C/EBPα and PPARγ [24]. In our study, although miR-6402 was identified as a negative regulator of adipocyte differentiation by suppressing C/EBPβ and PPARγ expression by targeting post-transcriptional regulation of *Bmpr2*, miR-6402 overexpression alone did not suppress the expression of *Pparg*, as shown in Figure 2(a). Therefore, suppression of the adipogenesis marker is a consequence of the direct targeting of the 3′UTR of *Bmpr2*, which precludes the post-transcriptional regulation of *Pparg*. These findings indicate a new signalling pathway in which miR-6402 influences adipogenesis or obesity by regulating BMP4, which activates BMPR2 and induces adipogenesis.

Although TNF-α is known to disrupt the adipogenesis process, our results demonstrated that TNF-α stimulation induced the expression of BMPR2, a receptor involved in adipogenesis. This phenomenon can be explained by the observed suppression of miR-6402 expression following TNF-α stimulation, which resulted in decreased degradation of the *Bmpr2* target gene and subsequent upregulation of BMPR2.

Previous studies have reported that BMP4 is upregulated in obese mice [25] and individuals [26]. However, BMP4 has also been reported to increase energy expenditure [27]. Thus, BMP4 May be involved in both the promotion and suppression of obesity. Our study demonstrated the inhibitory effect of miR-6402 on BMP4-induced adipogenesis. However, the possibility that miR-6402 promotes obesity cannot be ruled out, considering the results of previous studies wherein miR-21 expression was significantly suppressed one week after mice were fed an HFD and steadily increased during HFD feeding [28]. Further research is required to determine the dynamics of miR-6402 expression in long-term obesity.

## Conclusions

Our results indicated that miR-6402 plays an important role in the pathophysiology of obesity by suppressing BMP4-induced adipogenesis. Furthermore, miR-6402 can serve as a novel therapeutic agent for obesity and its associated metabolic complications by targeting *Bmpr2* as a signalling regulator of adipogenesis, rather than commonly studied adipogenesis markers such as *Pparg*.

## Materials and methods

### Mice

Forty-five mice were purchased from Jackson Laboratory (Bar Harbor 000664). Healthy male C57BL/6J mice with a body weight of 19–22 g at 6-week-old were selected as the study objects. They were placed in a specific pathogen-free animal feeding room with good ventilation and stable humidity, and had free access to food and water. Mice were housed in groups of 4–5 per cage. Animal centre staff monitored mice twice daily. Health was monitored by weight, food and water intake, and mice activity. After 2 weeks of adaptive feeding, the experiments were performed.

For a diet-induced obesity model, 14 mice were randomized into two groups and fed either ND (5 kcal% fat; Oriental Yeast Co., MF) or HFD (60 kcal% fat; Oriental Yeast Co., HFD-60) *ad libitum* for 8 weeks. The exclusion criterion in obesity model mice is less than 32 g of body weight. At 16 weeks of age, cervical dislocation was performed by an expert for euthanasia. Then, the eWAT was obtained. For RNA or protein analyses, we used eWATs from three or four mice, respectively.

For the *in vivo* adipogenesis study, mice were injected a triple anaesthetic mixture (medetomidine hydrochloride 0.75 mg/kg, midazolam 4 mg/kg, butorphanol tartrate 5 mg/kg), and then BMP4 (50 pg/g; Thermo Fisher Scientific, 315–27), atelocollagen (AteloGene local use, Koken, 1490), and control miRNA (2.7 nmol/g) or miR-6402 mimic (2.7 nmol/g) complexes were injected into the eWATs of 8-week-old mice. The left or right side eWATs were injected with BMP4 and control miRNA or BMP4 and miR-6402, respectively. Trypan blue staining was used to confirm the injection area. To ensure blinding, injection, sample collection, and subsequent sample analysis were performed by different experimenters. 24 h after injection, cervical dislocation was performed by an expert for euthanasia. The expression of miR-6402 in eWATs was analysed. eWATs from two mice were used for immunohistochemical staining and HE staining. eWATs from five mice were used for western blotting. miR-6402 (5‘-UCACCGGGUGUAAAUCAGCUUG-3’) and scrambled small RNA (control miRNA; 5‘-GAGCAGUUUUCCCAGGAACCCGC-3’) were synthesized by Ajinomoto Bio-Pharma Services.

The following parameters were assessed: microRNA, mRNA, and protein expression, adipocyte size and number. We used ImageJ to determine adipocyte size and number. The primary outcome of this study was miR-6402 function in adipogenesis. To statistically examine this, the number of mice shown above was used.

All experiments were performed three times to confirm reproducibility. The study protocols were approved by the Institutional Animal Care and Use Committee of Hiroshima University (permission number: A22–65 for HFD-feeding experiments), Kyushu University (permission number: A23-454-0 for BMP4/miR6402 injection experiments), and the ARRIVE guidelines 2.0 were followed.

### Cell culture

The murine preadipocyte cell line 3T3-L1 was obtained from the American Type Culture Collection (ATCC, CL-173). 3T3-L1 cells were maintained in Dulbecco’s modified Eagle’s medium (DMEM; Nacalai Tesque 08458–16) containing 10% bovine serum (BS; Thermo Fisher Scientific 16170–060) and 1% penicillin-streptomycin (P/S; Nacalai Tesque 09367–34) [29]. When the 3T3-L1 cells reached confluence, they were induced to differentiate into adipocytes. Differentiation was induced by treating cells with differentiation medium [DMEM containing with 10% foetal bovine serum (FBS; Sigma Aldrich 10270–106), 1% P/S, 0.5 mm 3-isobutyl-1-methylxanthine (Sigma Aldrich 28822-58-4), 1 μM dexamethasone (Sigma Aldrich 90357), and 10 μg/mL insulin (Cell Science & Technology Institute, 0105)]. After 2 days, the medium was replaced with post-differentiation medium (DMEM containing 10% FBS, 1% P/S, and 10 μg/mL insulin) to promote adipose formation, with daily replacement until day 7. For the experiments using differentiated 3T3-L1 cells, TNF-α (5 ng/mL, BioLegend 575202) was added on day 7.

The murine macrophage cell line RAW264.7 was obtained from ATCC. RAW264.7 cells were maintained in Roswell Park Memorial Institute 1640 medium (RPMI1640; Nacalai Tesque 30264–56) containing 10% FBS and 1% P/S.

Co-culture of 3T3-L1 and RAW264.7 cells was performed using a transwell plate with a 0.4-μm porous membrane insert (Corning Inc., Corning, 3412). Briefly, after differentiation of 3T3-L1 cells in the lower chamber, RAW264.7 cells were cultured in the upper chamber. The upper chamber medium contained *Escherichia coli* LPS (1 ng/mL; Sigma Aldrich, L6529) [30].

### RNA isolation and qPCR

Total RNA was isolated using ISOGEN II (Nippon Gene, 311–07361), in accordance with the manufacturer’s protocol. RNA purity was measured using NanoDrop Lite (Thermo Fisher Scientific 14109724–0). For mRNA and miRNA quantification, total RNA was reverse-transcribed to cDNA using a reverse transcription kit (Toyobo, FSQ-201) or an miRNA cDNA synthesis kit (Norgen Biotek Corp 54410). Taq-Pro UNI-SYBR qPCR reagent (Nippon Genetics, Q712–02) was used for qPCR analysis using the StepOnePlus system (Thermo Fisher Scientific 4376598). Glyceraldehyde-3-phosphate dehydrogenase (*GAPDH*) and U6 small nuclear 6 (*RNU6-6P*) were used as endogenous controls for mRNA and miRNA expression, respectively. The primer sequences used in this study are listed in Supplementary Table S2.

### Protein isolation and western blotting

Cells and tissues were harvested, washed twice with ice-cold PBS, and lysed in radioimmunoprecipitation assay (RIPA) buffer (Fujifilm Wako Pure Chemical, 188–02453) containing a protease inhibitor cocktail (Nacalai Tesque 25955–11). The tissues were homogenized on ice. Cell lysates and homogenized tissue were centrifuged at 10,000 × *g* for 20 min at 4°C. After extraction, the protein concentrations were quantified using a BCA protein assay kit (Fujifilm Wako Pure Chemical, 297–73101). An equal amount of total protein lysate was loaded onto each lane of sodium dodecyl sulphate-polyacrylamide gel for electrophoresis. BLUE Star PLUS Prestained Protein-Ladder (Nippon Genetics, NE-MWP04) was used. The transferred membranes were blocked with blocking reagent (Blocking One; Nacalai Tesque 03953–95) for 1 h at room temperature (RT) and then incubated with the primary antibodies for 1 h at RT or overnight at 4°C, followed by probing with secondary antibodies for 1 h at RT. All antibodies used in this study are listed in Supplementary Table S3. Proteins were detected using the ImageQuant LAS 4000 mini.

### Preparation of mimics and transient transfection

3T3-L1 cells were seeded, and at 50%–70% confluence, the control or miR-6402 mimic was transfected into cells using an siRNA/miRNA transfection reagent (INTERFERin; Polyplus 101000028), in accordance with the manufacturer’s instructions. Transfection was validated after 24 h by assessing the levels of miR-6402 using qPCR.

### Statistical analysis

Statistical analyses were performed using JMP Pro 17 software. Student’s *t*-test was used to compare two groups and Tukey – Kramer HSD test was used to compare multiple-group. Values are presented as mean ± SD. Statistical significance was defined by *p* < 0.05.

## Supplementary Material

Supplementary Tables.docx

Supplementary Figure legend and Alt_text.docx

## Data Availability

The data that support the findings of this study are available at Figshare (doi: 10.6084/m9.figshare.28200683). The obtained miRNA expression profile data in previous study [12] were deposited to the Gene Expression Omnibus database in NCBI (GSE accession: GSE216923).

## Abbreviations

BMP: bone morphogenetic protein
BMPR2: bone morphogenetic protein receptor type 2
C/EBPβ: CCAAT enhancer binding protein beta
HE: haematoxylin-eosin
HFD: high-fat diet
LPS: lipopolysaccharide
miRNA: microRNA
ND: normal diet
PPARγ: peroxisome proliferator-activated receptor gamma
qPCR: quantitative polymerase chain reaction
TNF-α: tumour necrosis factor-alpha
eWAT: epididymal white adipose tissue
