# Supplementary material for: miR-6402 targets Bmpr2 and negatively regulates mouse adipogenesis
Source: Adipocyte. 2025 Mar 3;14(1):2474114. doi: 10.1080/21623945.2025.2474114 (PMC11881869; doi:10.1080/21623945.2025.2474114)
Supplement: Supplementary Tables.docx [file KADI_A_2474114_SM4073.docx]

Supplementary Table S1. List of miRNAs reported their function in obesity.

| miRNAs | Function | Target | Reference |
| --- | --- | --- | --- |
| miR-144-3p | Prevents insulin resistance | *Irs1* | a |
| miR-378a-3p | Prevents obesity | *Yy1* | b |
| miR-193a-3p | Regulates insulin signaling | *Pten, Tsc2* | c |
| miR-130a-3p | Attenuates glucose intolerance | Phlpp2 | d |
| miR-107-3p | Impairs glucose intolerance | *Fasn* | e |
| miR-103-3p | Promotes insulin resistance | *Glut-4, Irs1, and Cav-1* | f |
| miR-30a-5p | Promotes insulin sensitivity | *Sirtuin1* | g |
| miR-145a-5p | Reduces insulin signaling | PI3K/Akt | h |
| miR-23b-3p | Promotes insulin resistance | *Neu1* | i |
| miR-23a-3p | Prevents insulin resistance | *Pten, S6k* | j |
| miR-143-3p | Promotes insulin resistance | *Igf2r* | k |
| miR-21a-5p | Increases insulin glucose uptake | *Pten* | l |
| miR-26a-5p | Prevents insulin secretion | *Rnf6* | m |

a. Dwi S Karoline, et.al. PLoS One, 2011, 6(8)

b. Jingjing Du N, et.al. Gut Microbes, 2021, 13(1):1–19

c. Ewa Ocłoń, et.al. 2016. J Endocrino., 229(3):259–66

d. Jiahan Wu, et.al. Metabolism, 2020, 103:154006.

e. H Bhatia, et.al. Int. J. Obes., 2016, 40(5):861–9.

f. Flávia de T Frias, et.al. J Cell Physiol., 2018, 233(4):3515–28

g. Shanshan Cui, et.al. Int. J. Clin. Exp. Pathol., 2018, 11(11):5203–12

h. Li Wang, et.al. Genomics, 2020, 112(4):2688–94

i. Yan Wei, et.al. BMC Endocr Disord., 2021, 21(1):57

j. Javier Lozano-Bartolomé, et.al. J. Clin. Endocrinol. Metab., 2018, 103(4):1447–58

k. Lin Xihua, et.al. Transl Res., 2019, 205:33–43

l. H-Y Ling, et.al. Exp Clin Endocrinol Diabetes, 2012, 120(9):553–9

m. Fan Yang, et.al. Diabetes Metab Syndr Obes., 2022, 15:93–102

Supplementary Table S2. Sequence of primers used in this study.

| Gene | Forward | Reverse |
| --- | --- | --- |
| *Gapdh* | AATGTGTCCGTCGTGGATCTGA | GATGCCTGCTTCACCACCTTCT |
| *Bmpr2* | TTGGGATAGGTGAGAGTCGAAT | TGTTTCACAAGATTGATGTCCCC |
| *Cpt1a* | CCAGGCTACAGTGGGACATT | GAACTTGCCCATGTCCTTGT |
| *Tmem26* | GAAACCAGTATTGCACCC | CCCATTCCATTGGTGGCTCT |
| *Traf6* | ACTGGGGACAATTCACTAGAGC | AAAGCGAGAGATTCTTTCCCTG |
| *Pparg* | CCAGAGTCTGCTGATCTGCG | GCCACCTCTTTGCTCTGCTC |
| *Acox1* | TCCAGACTTCCAACATGAGGA | CTGGGCGTAGGTGCCAATTA |
| *Irak4* | CCTGGATGTCCTGGAACTTG | CAACACGCAGTAGGCAGAGA |
| *AdipoR2* | GCCCAGCTTAGAGACACCTG | GCCTTCCCACACCTTACAAA |
| *Sirt1* | GCATAGATACCGTCTCTTGATCTGAA | TGTGAAGTTACTGCAGGAGTGTAAA |
| *Cxcl1* | CACCCAAACCGAAGTCATAG | AAGCCAGCGTTCACCAGA |
| *Adiponectin* | AAGGACAAGGCCGTTCTCT | CGCACGATTTCCCTCTCAGCTG |
| *Flcn* | GCACCCAGGCTATATCAGTCA | GACCAGGGCATACCTCACA |
| *G3bp1* | TTGAGGACGTTTTCTTGGGCA | CTGTGACGCTGGCACTTTG |
| *Exoc6b* | CTGCCACTGTCGATAAGCTAATG | GCAGATAGGTGTGTTCCAGATG |
| *Gadd45a* | AGACCGAAAGGATGGACACG | GTACACGCCGACCGTAATG |
| *C/ebpβ* | CAAGCTGAGCGACGAGTACA | AGCTGCTCCACCTTCTTCTG |

*Gapdh*: glyceraldehyde-3-phosphate dehydrogenase; *Bmpr2*: bone morphogenetic receptor type 2; *Cpt1a*: carnitine palmitoyl transferase-type 1 alpha; *Tmem26*: transmembrane protein 26; *Traf6*: TNF receptor-associated factor 6*; Pparg*: peroxisome proliferator-activated receptor gamma; *Acox1*: acyl-CoA oxidase 1; *Irak4*: interleukin-1 receptor-associated kinase 4; *AdipoR2*: adiponectin receptor 2; *Sirt1*: sirtuin 1; *Cxcl1*: C-X-C motif chemokine ligand 1; *Flcn*: folliculin; *G3bp1*: GTPase-activating protein-binding protein 1; *Exoc6B*: exocyst complex component 6B; *Gadd45a*: growth arrest and DNA damage inducible alpha; *C/ebpβ*: CCAAT enhancer binding protein beta

Supplementary Table S3. List of antibodies used in western blotting.

| Antibody (Catalog number) | Source |
| --- | --- |
| β-actin (A5441)  BMPR2 (19087-1-AP) | Sigma Aldrich, Saint Louis ProteinTech, Rosemont |
| C/EBPβ (LAP) #3087  PPARγ (81B8) #2443 | Cell Signaling Technology  Cell Signaling Technology |

BMPR2: bone morphogenetic receptor type 2; C/EBPβ: CCAAT enhancer binding protein beta; PPARγ: peroxisome proliferator-activated receptor gamma
