# Supplementary material for: miR-6402 targets Bmpr2 and negatively regulates mouse adipogenesis
Source: Adipocyte. 2025 Mar 3;14(1):2474114. doi: 10.1080/21623945.2025.2474114 (PMC11881869; doi:10.1080/21623945.2025.2474114)
Supplement: Supplementary Figure legend and Alt_text.docx [file KADI_A_2474114_SM4072.docx]

**Supplementary Fig. S1.** (a) Changes in the body weight of ND- and HFD-fed mice. (b) *Tnfa* and *Mcp-1* expression of ND- and HFD-fed mice analyzed by qPCR analysis. (c) miR-6402 expression in preadipocytes (undifferentiated 3T3-L1 cells), adipocytes (differentiated 3T3-L1 cells), and RAW264.7 macrophage analyzed by qPCR analysis. The graphs represent the mean ± S.D. (n = 3 for each group). **p* < 0.05, ***p* < 0.01, ****p* < 0.001 between the two groups (a and b; Student’s *t*-test; c, Tukey–Kramer HSD test).

**Alt-text** **of** **Supplementary Fig. S1.** (a) HFD-fed mice were significantly higher in their body weight compared to ND-fed mice. (b) *Tnfa* and *Mcp-1* gene expression were upregulated in eWATs of HFD-fed mice. (c) miR-6402 expression was the lowest in RAW264.7 macrophages compared to 3T3-L1 and differentiated 3T3-L1 cells.

**Supplementary Fig. S2.** Heatmap showing the expression levels of the top 100 miRNAs with high expression levels in WAT of ND-fed mice among the miRNAs detected by microarray analysis (left line of the heatmap). The corresponding data in WAT of HFD-fed mice are shown on the right side. Data of HFD-fed mice, which are lower than that of ND-fed mice, are marked with an asterisk. Eighteen miRNAs were selected because the ratio is less than 0.45 in comparison with ND-fed mouse value.

**Alt-text** **of** **Supplementary Fig. S2.** Heatmap showing the expression levels of the top 100 miRNAs with high expression levels in WAT of ND-fed mice among the miRNAs detected by microarray analysis (left line of the heatmap).

**Supplementary Fig. S3.** qPCR analysis showing the expression of miR-6402 in undifferentiated (a) and differentiated (b) 3T3-L1 cells. The bar graphs represent the mean ± S.D. (n = 3 for each group). ***p* < 0.01, ****p* < 0.001 between the indicated bars of the two groups (Student’s *t*-test).

**Alt-text of Supplementary Fig. S3**. (a,b) miR-6402 expression was successfully upregulated after miR-6402 transfection in 3T3-L1 and differentiated 3T3-L1 cells.

**Supplementary Fig. S4.** Original whole-membrane images of Fig. 2b. BMPR2 expression in undifferentiated 3T3-L1 cells transfected with Control and miR-6402 mimics was analyzed by western blotting. Arrowheads represent each detected band. Molecular weights in kDa are shown on the left.

**Alt-text of Supplementary Fig. S4.** Original western blot whole-membrane images of Fig. 2b.

**Supplementary Fig. S5.** Original whole-membrane images of Fig. 2d. BMPR2 expression in differentiated 3T3-L1 cells transfected with Control and miR-6402 mimics was analyzed by western blotting. Arrowheads represent each detected band. Molecular weights in kDa are shown on the left.

**Alt-text of Supplementary Fig. S5.** Original western blot whole-membrane images of Fig. 2d.

**Supplementary Fig. S6.** Original and repeated images of Fig. 2f. BMPR2 expression in undifferentiated 3T3-L1 cells stimulated with TNF-α was analyzed by western blotting. The original whole-membrane images of Fig. 2f are shown (upper panels). The data of three repeated experiments (Repeat 1, Repeat 2, and Repeat 3) are shown. Arrowheads represent each detected band. Molecular weights in kDa are shown on the left.

**Alt-text of Supplementary Fig. S6.** Original and repeated western blot images of Fig. 2f.

**Supplementary Fig. S7.** Original and repeated images of Fig. 2h. BMPR2 expression in differentiated 3T3-L1 cells stimulated with TNF-α was measured by western blotting. The original whole-membrane images of Fig. 2h are shown (upper panels). The data of three repeated experiments (Repeat 1, Repeat 2, and Repeat 3) are shown. Arrowheads represent each detected band. Molecular weights in kDa are shown on the left.

**Alt-text of Supplementary Fig. S7.** Original and repeated western blot images of Fig. 2h.

**Supplementary Fig. S8.** Original whole-membrane images of Fig. 3d. C/EBPβ and PPARγ expression in WAT of ND- or HFD-fed mice was analyzed by western blotting. Arrowheads represent each detected band. Molecular weights in kDa are shown on the left.

**Alt-text of Supplementary Fig. S8.** Original whole-membrane western blot images of Fig. 3d.

**Supplementary Fig. S9.** For the *in vivo* adipogenesis study, miR-6402 expression levels in eWATs of each mouse (No. 1–7) were measured by qPCR. The left or right side eWATs were injected with BMP4 and control miRNA (-) or BMP4 and miR-6402 (+), respectively.

**Alt-text of Supplementary Fig. S9.** miR-6402 expression was successfully upregulated after miR-6402 injected in eWATs of all mice.

**Supplementary Fig. S10.** Other images of Fig. 4a. BMP4 was injected into WAT together with control mimic or with miR-6402 mimic. The images of BMPR2 immunohistochemical staining (a) and hematoxylin-eosin staining (b) are shown.

**Alt-text of Supplementary Fig. S10.** Additional images of BMPR2 immunohistochemical staining (a) or hematoxylin-eosin staining (b) of Fig. 4a.

**Supplementary Fig. S11.** Original whole-membrane images of Fig. 4e. BMP4 was injected into WAT together with control mimic or with miR-6402 mimic. Arrowheads represent each detected band. Molecular weights in kDa are shown on the left.

**Alt-text of Supplementary Fig. S11.** Original whole-membrane western blot images of Fig. 4e.
